# Investigation of the relationship of CO-RADS and CT patterns with laboratory parameters in COVID-19 patients and a new perspective on the total CT scoring system

**DOI:** 10.1186/s12880-022-00857-8

**Published:** 2022-07-20

**Authors:** Nevin Aydin, Pinar Yildiz, Döndü Üsküdar Cansu, Elif Gündogdu, Rüya Mutluay, Göknur Yorulmaz, Melisa Sahin Tekin, Evin Kocaturk, İ. Özkan Alatas, Elif Doyuk Kartal, Nurettin Erben, Gül Durmaz, Nilgun Kasifoglu, Tercan Us, Garip Sahin, Cengiz Bal, Senay Yilmaz, Cengiz Korkmaz

**Affiliations:** 1 Department of Radiology, Faculty of Medicine, Eskisehir Osmangazi University, 26040 Eskisehir, Turkey; 2 Department of Internal Medicine, Faculty of Medicine, Eskisehir Osmangazi University, Eskisehir, Turkey; 3 Department of Biochemistry, Faculty of Medicine, Eskisehir Osmangazi University, Eskisehir, Turkey; 4 Department of Infectious Diseases and Clinical Microbiology, Faculty of Medicine, Eskisehir Osmangazi University, Eskisehir, Turkey; 5 Department of Medical Microbiology, Faculty of Medicine, Eskisehir Osmangazi University, Eskisehir, Turkey; 6 Department of Biostatistics, Faculty of Medicine, Eskisehir Osmangazi University, Eskisehir, Turkey; 7 Department of Chest Diseases, Faculty of Medicine, Eskisehir Osmangazi University, Eskisehir, Turkey

**Keywords:** CT, CO-RADS, CT pattern, COVID-19, HRCT, Total CT score

## Abstract

**Background:**

It is important to determine the correlation of the CO-RADS classification and computed tomography (CT) patterns of the lung with laboratory data. To investigate the relationship of CO-RADS categories and CT patterns with laboratory data in patients with a positive RT-PCR test. We also developed a structured total CT scoring system and investigated its correlation with the total CT scoring system.

**Method:**

The CT examinations of the patients were evaluated in terms of the CO-RADS classification, pattern groups and total CT score. Structured total CT score values were obtained by including the total CT score values and pattern values in a regression analysis. The CT data were compared according to the laboratory data.

**Results:**

A total of 198 patients were evaluated. There were significant differences between the CO-RADS groups in terms of age, ICU transfer, oxygen saturation, creatinine, LDH, D-dimer, high-sensitivity cardiac troponin-T (hs-TnT), CRP, structured total CT score values, and total CT score values. A significant difference was also observed between the CT pattern groups and oxygen saturation, creatinine and CRP values. When the structured total CT score values and total CT score values were compared they were observed to be correlated.

**Conclusions:**

Creatinine can be considered as an important marker for the CO-RADS and pattern classifications in lung involvement. LDH can be considered as an important marker of parenchymal involvement, especially bilateral and diffuse involvement. The structured total CT scoring system is a new system that can be used as an alternative.

## Background

The coronavirus disease 2019 (COVID-19), declared as a pandemic by the World Health Organization (WHO). The radiological imaging of the lung has gained importance in terms of early diagnosis, follow-up and treatment of COVID-19 disease [1–3]. The reverse transcription-polymerase chain reaction (RT-PCR) test is used as a reference method for the early diagnosis of the disease [4].

The COVID-19 reporting and data system (CO-RADS) is a standardized system for COVID-19 computed tomography (CT) [5]. The lung parenchyma involvement pattern may be in the form of ground glass opacities, areas of consolidation, or both. The underlying histological patterns of these findings have been observed as vascular damage, diffuse alveolar damage, and thrombosis [6].

In COVID-19 pneumonia, a scoring system calculated for each lobe in all lung lobes is also used both in Chest X-Ray and in chest CT. In this scoring system, the prevalence in the distribution of lesions is kept in the foreground in the evaluation, and the characterization of the lesions is not taken into account [7–9]. However, there is still a need for scoring systems that include the lung CT parenchymal involvement pattern [10]. For this purpose, it is important to determine the correlation of the CO-RADS classification and CT patterns of the lung with laboratory data. In a few studies, it was found that the laboratory parameters of D-dimer, lactate dehydrogenase (LDH), procalcitonin and C-reactive protein (CRP), as well as lung CT scoring showed the prognosis of patients with COVID-19 pneumonia [11].

In this study, we aimed to investigate the correlation of the CO-RADS category and lung CT patterns with laboratory data in patients with a positive RT-PCR test for COVID-19. In addition, we developed a total CT scoring system by including the patients’ CT patterns in a regression analysis and investigated its relationship with the total CT scoring system.

## Methods

### Patient population

**Fig. 1:**
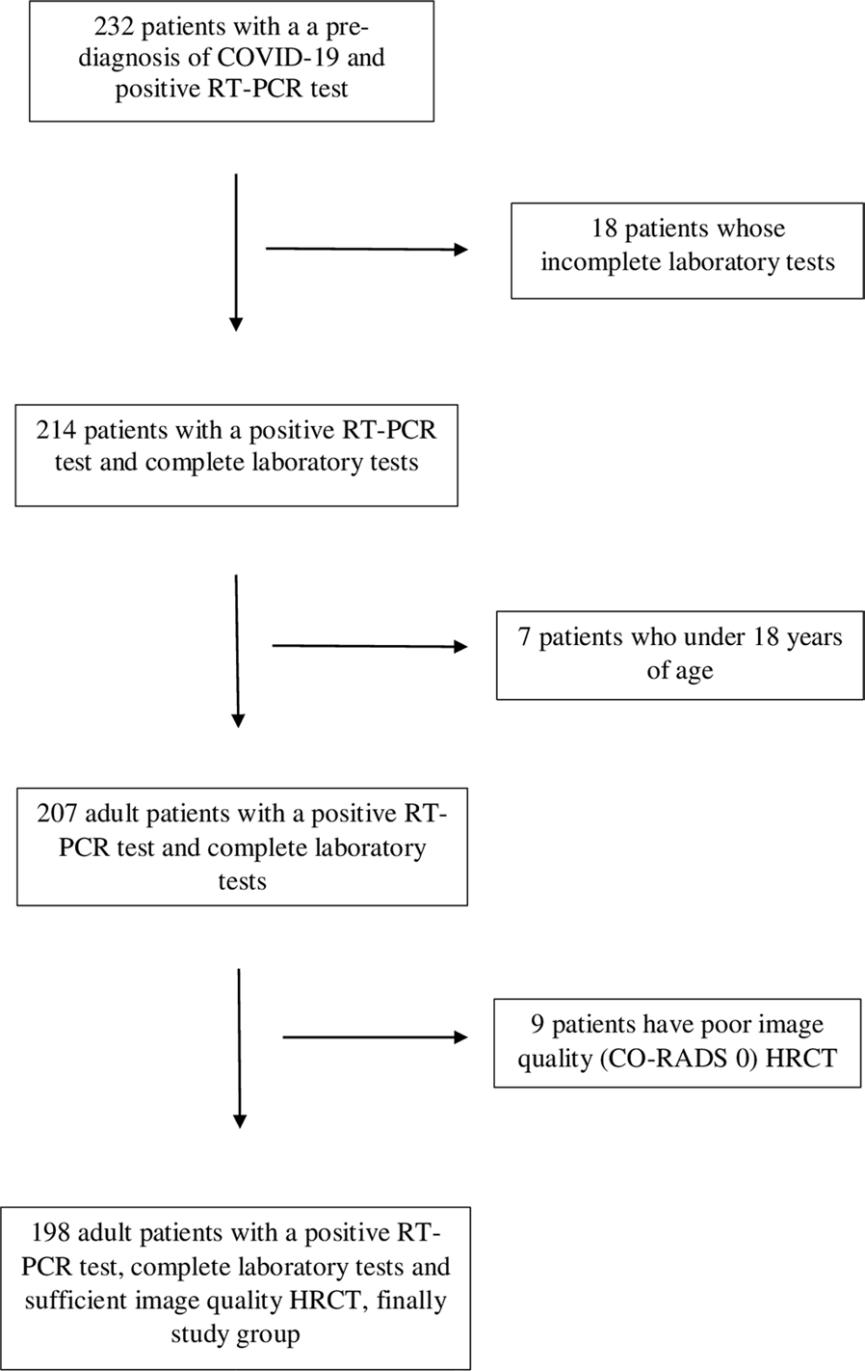
Flow chart of the patient population

Patients who presented to our hospital with a pre-diagnosis of COVID-19 between March 2020 and August 2020 were retrospectively evaluated. Patients diagnosed with COVID-19 based on a positive RT-PCR test, who underwent laboratory tests and high-resolution CT (HRCT) were included in the study. Those with, an incomplete laboratory tests and under 18 years of age were excluded from the study. And also CORADS 0 patient group was excluded. Detailed patient information was mentioned in the flow chart (Fig. 1).

The patients’ demographic data, detailed symptoms and findings of COVID-19, chronic disease status, intensive care unit (ICU) transfer, and one-month survival were recorded. The patients were also evaluated in detail in terms of CT findings, CO-RADS classification, pattern groups, and total CT scoring.

### Imaging procedure

HRCT scans were performed using a 64-slice CT scanner (Siemens, SOMATOM 2015) at a section thickness of 0.6 mm. HRCT images were evaluated in the parenchymal window. HRCT examinations were performed with a window width of 1400 and window height of − 400 at 100 kV and 243 mA. In the parenchymal window, images with a matrix size of 672 × 672 pixels were used.

### Imaging analysis

Images were evaluated by two radiologists with nine years (E.G.) and seven years (N.A.) of experience. All HRCT scans were evaluated separately by both radiologists. Cases with disagreement after the initial evaluation were re-evaluated by consensus by two radiologists. At the points where the disagreement persisted, the results of the more experienced radiologist in thoracic imaging (N.A) were used for statistical analysis in these cases. The radiologists evaluated the patients’ HRCTs without knowing their clinical and laboratory findings. The HRCT examinations were classified according to the CO-RADS classification. CO-RADS groups 1, 3, 4 and 5 were included in the study. Since all the patients were positive for RT-PCR, CO-RADS categories 2 and 6 were excluded from the study. The tomography examinations with poor imaging quality (CO-RADS 0) were excluded from the study. The HRCT patterns of the patients were grouped according to the presence of normal findings (0), ground glass opacities (1), ground glass opacities + consolidation (mixed pattern) (2), and consolidation (3). The consolidation pattern was not evaluated in the study since there was no patient with this pattern alone. The HRCT examinations were scored according to the CT scoring system based on lobe involvement as follows: 0 point was given if there was no lobe involvement, 1 point if < 5% involvement, 2 points if 5–25% involvement, 3 points if 26–50% involvement, 4 points if 51–75% involvement, and 5 points if 75% involvement [7]. The structured total CT score was obtained by including the CT score values and pattern values in a regression analysis. In addition to the imaging of the patients, the following laboratory parameters were evaluated at first visit: hemoglobin, white blood cell (WBC), absolute neutrophil count (ANC), absolute lymphocyte count (ALC), creatinine, LDH, D-dimer, high-sensitivity cardiac troponin-T (hs-TnT), CRP and oxygen saturation.

### Statistical analysis

Continuous data were given as mean ± standard deviation and median (Q1-Q3) values. Categorical data were given as percentages (%) and numbers. The Shapiro–Wilk test was used to investigate the conformance of the data to the normal distribution. In the comparison of normally distributed variables, the independent-samples t-test was used for two groups and one-way analysis of variance for three or more groups. In the comparison of non-normally distributed variables, the Mann–Whitney U test was used for two groups and the Kruskal–Wallis H test for three or more groups. Pearson’s correlation coefficient was calculated for the normally distributed variables, and Spearman’s correlation coefficient for those without a normal distribution. The Pearson chi-square and Pearson exact chi-square analyses were conducted to further examine the created cross tables. In the estimation of the structured score value, modeling was performed by calculating the beta coefficients with the linear regression analysis method.

The intraclass correlation coefficient (ICC) was used to assess interobserver variability. Based on the 95% confidence interval (CI) of the ICC estimate, values less than 0.5, 0.5 to 0.75, 0.75 to 0.9, and greater than 0.90 indicate poor, moderate, good, and excellent reliability, respectively.

IBM SPSS Statistics v. 21.0 (IBM Corp. Released 2012. IBM SPSS Statistics for Windows, Version 21.0. Armonk, NY: IBM Corp.) was used in statistical analyses. A p value of < 0.05 was accepted as a criterion for statistical significance.

This study was conducted retrospectively, and all patients provided signed consent before undergoing HRCT. Ethics committee approval was received on June 1, 2021 (decision number: 06, E-25403353-050.99-197067).

## Results

A total of 198 patients with a positive RT-PCR test who met the inclusion criteria were evaluated. The mean age of the patients was 46.3 ± 17.1 years. The patients’ descriptive characteristics, chronic diseases, and radiological imaging categories are given in Table 1 in detail.

**Table 1:** Patients’ descriptive characteristics, chronic diseases and radiological ımaging categories

	Total (n = 198)
Age, years	46.3 ± 17.1
	44.5 (32.0–57.8)
Gender	
Male	94 (47.5%)
Female	104 (52.5%)
Chronic disease	
Hypertension	37 (18.7%)
Malignancy	8 (4.04%)
Diabetes mellitus	22 (11.1%)
Kidney disease	3 (1.52%)
Smoking	17 (8.59%)
Known lung disease	35 (17.69%)
Coronary artery disease	6 (3.03%)
Heart failure	2 (1.01%)
Rhythm disorder	1 (0.50%)
Rheumatological disease	3 (1.52%)
Oxygen saturation	96.6 ± 2.99
	98.0 (96.0–98.0)
Radiological ımaging	
CO-RADS	
1	127 (64.1%)
3	15 (7.6%)
4	20 (10.1%)
5	36 (18.2%)
Pattern	
Pattern 0	127 (64.1%)
Pattern 1	53 (26.8%)
Pattern 2	18 (9.09%)
Structured total CT score	7.72 ± 3.39
	7.00 (5.00–10.0)
Total CT score	4.80 ± 3.75
	4.00 (2.00–7.00)

In the evaluation of HRCT image, ICC (95% CI) indicated good agreement (0.85) between the first and second radiologists.

**Fig. 2:**
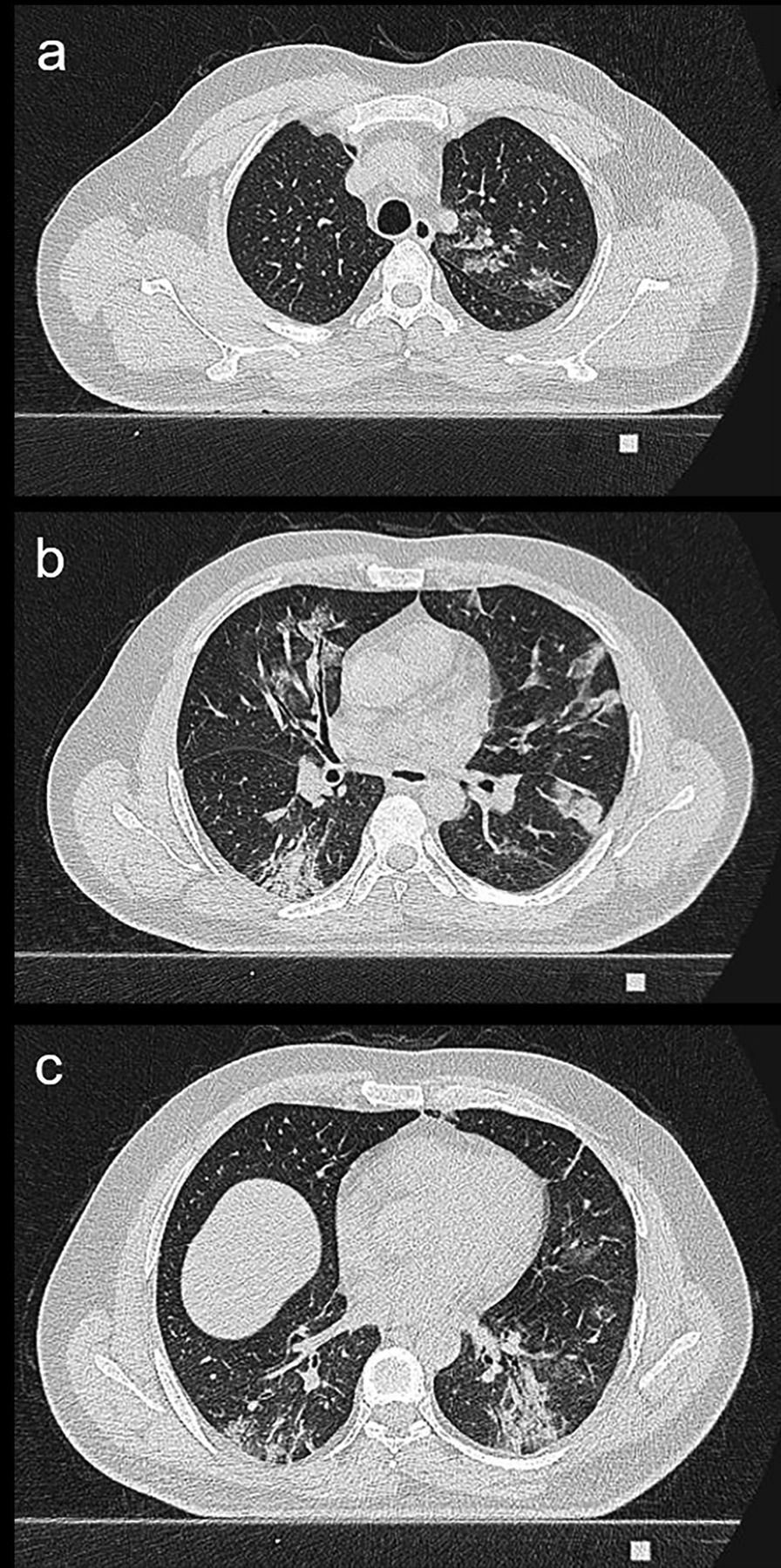
65 years old male patient. According to the CO-RADS category in terms of Covid pneumonia, a CO-RADS 5 patient had a mixed parenchyma pattern (pattern 2), and the patient's total CT score value was 11 and the structured total CT score value was 22 according to **a**, **b** and **c**

**Fig. 3:**
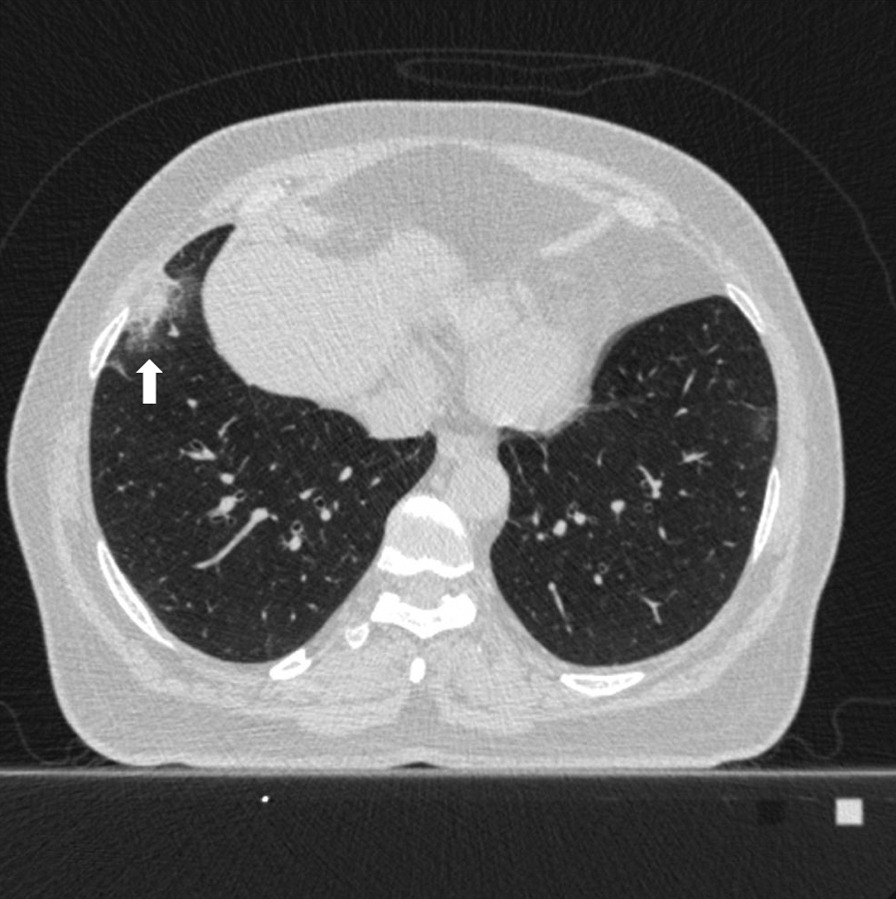
48 years old female patient. According to the CO-RADS category in terms of Covid pneumonia, a CO-RADS 4 patient had a mixed parenchyma pattern (pattern 2) with a peripheral lesion (white arrow), and the patient's total CT score value was 2 and the structured total CT score value was 4 according to HRCT scan

**Fig. 4:**
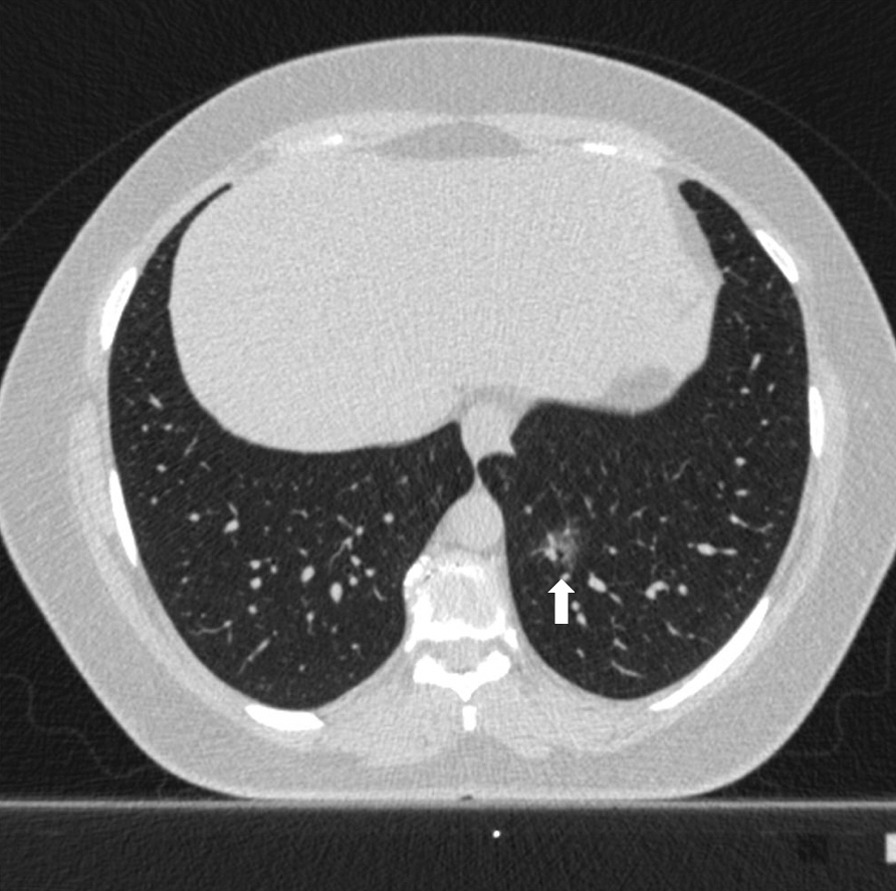
56 years old female patient. According to the CO-RADS category in terms of Covid pneumonia, a CO-RADS 3 patient had a ground glass parenchyma pattern (pattern 1) with a central lesion (white arrow), and the patient's total CT score value was 1 and the structured total CT score value was 1 according to HRCT scan

The beta value between the structured total CT score and the total CT score value was found to be significant at − 0.208. The structured total CT score was calculated according to the regression analysis with the following formula: − 0.208 + 1.474 * pattern + 0.947 * total CT score. Images of the patients belonging to CO-RADS 5, 4 and 3 and their score and structured score values were given in Figs. 2, 3 and 4, respectively.

Significant differences were found between the CO-RADS groups 1, 3, 4 and 5 in relation to age (p = 0.0319), ICU transfer (p = 0.029), oxygen saturation (p < 0.001), creatinine (p = 0.007), LDH (p = 0.013), D-dimer (p = 0.022), hs-TnT (p = 0.040), CRP (p < 0.001) (Table 2), structured total CT score values (p < 0.001) and total CT score values (p < 0.001) (Table 3).

**Table 2:** Relationship of the CO-RADS category with clinical and laboratory parameters

CO-RADS	1 (n = 127)	3 (n = 15)	4 (n = 20)	5 (n = 36)	p value
Age, years	45.0 ± 16.7	46.7 ± 24.3	42.7 ± 16.6	52.9 ± 14.2	0.032*
	42.0 (32.0–57.0)	38.0 (29.5–64.0)	45.0 (27.8–51.8)	48.0 (42.0–61.3)	
Gender					
Male	61 (48.0%)	8 (53.3%)	7 (35.0%)	18 (50.0%)	0.668
Female	66 (52.0%)	7 (46.7%)	13 (65.0%)	18 (50.0%)	
Intensive care unit transfer	5 (3.94%)	3 (20.0%)	0 (0%)	4 (11.1%)	0.029*
Survival	125 (98.4%)	14 (93.3%)	20 (100%)	34 (94.4%)	0.338
Oxygen saturation	97.5 ± 1.56	95.2 ± 6.07	96.0 ± 2.03	94.4 ± 3.99	< 0.001*
	98.0 (97.0–98.0)	96.0 (95.5–98.0)	96.0 (94.0–98.0)	94.5 (91.8–98.0)	
Hemoglobin	13.7 ± 1.88	14.2 ± 1.84	14.2 ± 1.52	13.5 ± 1.89	0.663
	13.8 (12.7–14.8)	13.9 (12.8–15.8)	13.9 (13.1–15.1)	13.5 (12.5–15.2)	
White blood cell	6.24 ± 2.07	6.73 ± 2.45	6.03 ± 2.09	5.33 ± 1.81	0.072
	5.92 (4.75–7.64)	6.10 (5.54–7.25)	5.75 (5.01–6.63)	5.13 (4.44–5.92)	
Absolute neutrophil count	3.66 ± 1.81	3.80 ± 1.84	3.59 ± 2.10	3.45 ± 1.78	0.757
	3.42 (2.45–4.67)	3.74 (2.48–4.24)	2.90 (2.43–3.97)	3.16 (2.44–4.00)	
Absolute lymphocyte count	1.72 ± 0.781	1.57 ± 0.698	1.73 ± 0.581	1.45 ± 0.759	0.213
	1.54 (1.16–2.20)	1.40 (1.20–2.05)	1.68 (1.32–2.28)	1.42 (0.900–1.76)	
Creatinine	0.895 ± 0.589	0.822 ± 0.247	0.838 ± 0.324	1.14 ± 0.655	0.007*
	0.800(0.650–0.943)	0.780(0.650–0.955)	0.770(0.625–0.900)	0.965 (0.743–1.17)	
Lactate dehydrogenase	203 ± 72.2	191 ± 64.4	202 ± 50.1	267 ± 131	0.013*
	194 (171–218)	190 (162–214)	183 (169–226)	217 (187–319)	
D-dimer	0.560 ± 0.956	0.613 ± 0.933	0.401 ± 0.289	1.49 ± 3.59	0.022*
	0.310(0.200–0.520)	0.260(0.190–0.360)	0.340(0.270–0.400)	0.540 (0.285–0.990)	
High-sensitivity cardiac troponin-T	0.0265 ± 0.183	0.00500 ± 0.00941	0.00462 ± 0.00660	0.0142 ± 0.0179	0.040*
	0 (0–0.0100)	0 (0–0.00750)	0 (0–0.0100)	0.0100(0.00250–0.0100)	
C-reactive protein	13.3 ± 38.5	22.0 ± 41.9	12.5 ± 25.6	41.6 ± 58.0	< 0.001*
	4.40(1.60–8.30)	7.70(3.75–17.40)	4.40(0.925–12.20)	20.60(7.80–45.00)	

p = 0.032*Significant difference in age between the CO-RADS groups 1 and 5 (p = 0.006) and groups 4 and 5 (p = 0.022)

p = 0.029*Significant difference in intensive care unit transfer between the CO-RADS groups 1 and 3 (p < 0.05)

p < 0.001*Significant difference in oxygen saturation between the CO-RADS groups 1 and 5 (p < 0.001) and CO-RADS groups 1 and 4

p = 0.007*Significant difference in creatinine between the CO-RADS groups 1 and 5 (p = 0.001) and CO-RADS groups 4 and 5 (p = 0.015)

p = 0.013* Significant difference in Lactate Dehydrogenase between the CO-RADS groups 3 and 5 (p = 0.014), CO-RADS groups 4 and 5 (p = 0.028), and groups 1 and 5 (p = 0.003)

p = 0.022*Significant difference in D-dimer between the CO-RADS groups 3 and 5 (p = 0.020) and CO-RADS groups 1 and 5 (p = 0.004)

p = 0.040*Significant difference in high-sensitivity cardiac troponin-T between the CO-RADS groups 3 and 5 (p = 0.042)

p < 0.001*Significant difference in C-reactive protein between the CO-RADS groups 1 and 5 and CO-RADS groups 4 and 5 (p < 0.001)

**Table 3:** Relationship of the CO-RADS category with the structured total CT score values and total CT score values

CO-RADS	1 (n = 127)	3 (n = 15)	4 (n = 20)	5 (n = 36)	p value
Structured total CT score values	–	4.93 ± 2.05	5.70 ± 1.34	10.00 ± 3.05	< 0.001*
		4.00 (4.00–5.00)	5.50 (5.00–7.00)	9.50 (8.00–12.0)	
Total CT score values	–	2.07 ± 2.31	3.25 ± 2.22	8.81 ± 4.08	< 0.001*
		1.00 (1.00–2.00)	2.50 (2.00–4.00)	8.00 (6.00–11.0)	

p < 0.001*significant difference in structured total CT score values between the CO-RADS groups 3 and 5 (p = 0.017)

p < 0.001*significant difference in total CT score values between the CO-RADS groups 3 and 5 (p = 0.015), and CO-RADS groups 4 and 5 (p = 0.036)

A significant difference was observed between the CT pattern groups 0, 1, and 2 in terms of oxygen saturation (p < 0.001), creatinine (p = 0.006) and CRP (p < 0.001) values. Apart from this, no correlation was found between CT patterns and clinical and laboratory parameters (Table 4). When the pattern groups were compared according to the total CT score values, no significant difference was found. Since the structured total CT score values were obtained from the pattern groups in the regression analysis, these scores were not compared according to the pattern group (Table 5).

**Table 4:** Relationship of the pattern groups with clinical and laboratory parameters

Pattern	0 (n = 127)	1 (n = 53)	2 (n = 18)	p value
Age, years	45.0 ± 16.7	50.5 ± 18.6	43.4 ± 14.0	0.151
	42.0 (32.0–57.0)	47.0 (38.0–66.0)	45.5 (32.5–55.3)	
Gender				
Male	61 (48.0%)	28 (52.8%)	5 (27.8%)	0.18
Female	66 (52.0%)	25 (47.2%)	13 (72.2%)	
Intensive care unit transfer	5 (3.94%)	6 (11.3%)	1 (5.56%)	0.166
Survival	125 (98.4%)	51 (96.2%)	17 (94.4%)	0.479
Oxygen saturation	97.5 ± 1.56	94.7 ± 4.47	96.0 ± 2.68	< 0.001*
	98.0 (97.0–98.0)	95.0 (94.0–98.0)	96.5 (95.0–98.0)	
Hemoglobin	13.7 ± 1.88	14.1 ± 1.77	13.1 ± 1.68	0.144
	13.8 (12.7–14.8)	14.3 (12.9–15.3)	13.2 (12.6–14.0)	
White blood cell	6.24 ± 2.07	5.95 ± 2.04	5.43 ± 2.21	0.295
	5.92 (4.75–7.64)	5.50 (4.85–6.40)	5.38 (4.19–6.49)	
Absolute neutrophil count	3.66 ± 1.81	3.67 ± 1.78	3.25 ± 2.11	0.42
	3.42 (2.45–4.67)	3.20 (2.54–4.15)	2.90 (2.12–3.87)	
Absolute lymphocyte count	1.72 ± 0.781	1.56 ± 0.635	1.53 ± 0.890	0.484
	1.54 (1.16–2.20)	1.44 (1.19–1.90)	1.50 (0.958–1.80)	
Creatinine	0.895 ± 0.589	1.06 ± 0.588	0.784 ± 0.208	0.006*
	0.800 (0.650–0.943)	0.905 (0.728–1.12)	0.705 (0.623–0.943)	
Lactate dehydrogenase	203 ± 72.2	246 ± 118	197 ± 49.7	0.081
	194 (171–218)	207 (175–279)	193 (155–223)	
D-dimer	0.560 ± 0.956	0.991 ± 2.78	0.988 ± 2.16	0.223
	0.310 (0.200–0.520)	0.365 (0.230–0.695)	0.375 (0.298–0.530)	
High-sensivity cardiac troponin	0.0265 ± 0.183	0.00868 ± 0.0138	0.0113 ± 0.0164	0.56
	0 (0–0.0100)	0.00500 (0–0.0100)	0.0100 (0–0.0100)	
C-reactive protein	13.3 ± 38.5	29.1 ± 42.1	29.9 ± 66.2	< 0.001*
	4.40 (1.60–8.30)	10.8(3.80–31.60)	10.3 (4.10–26.30)	

p < 0.001*Significant difference in oxygen saturation between the pattern groups 0 and 1 (p < 0.001), and the pattern groups 0 and 2 (p = 0.031)

p = 0.006*Significant difference in creatinine between the pattern groups 1 and 2 (p = 0.024), and the pattern groups 0 and 1 (p = 0.003)

p < 0.001*Significant difference in C-reactive protein between the pattern groups 0 and 1 (p < 0.001)

**Table 5:** Relationship of the pattern groups with the total CT score values

Pattern	0 (n = 127)	1 (n = 53)	2 (n = 18)	p value
Total CT score value	–	5.09 ± 4.04	3.94 ± 2.62	0.548
		4.00 (2.00–8.00)	3.50 (2.00–5.00)	

When the structured total CT score values and the total CT score values were compared according to the clinical data separately, significant differences were found in relation to the presence of malignancy (p = 0.012 and p = 0.016, respectively), known history of lung disease (p = 0.016 and p = 0.023, respectively), ICU transfer (p = 0.031 and p = 0.025, respectively), and survival (p = 0.010 and p = 0.011, respectively). Apart from this, no other significant relationship was observed with clinical data. The findings are given in Table 6 in detail.

**Table 6:** Relationship of the structured total CT score values and the total CT score values with clinical parameters

	Structured total CT score values			Total CT score values		
	Mean ± standard deviation		p value	Mean ± standard deviation		p value
	Male	Female		Male	Female	
Gender	7.93 ± 3.64	7.52 ± 3.18	0.672	5.21 ± 4.04	4.44 ± 3.49	0.422
	Present	Absent	p value	Present	Absent	p value
Hypertension	8.83 ± 4.09	7.34 ± 3.06	0.183	5.94 ± 4.60	4.41 ± 3.37	0.238
Malignancy	11.83 ± 4.30	7.33 ± 3.06	0.012	9.0 ± 4.69	4.41 ± 3.44	0.016
Diabetes mellitus	9.2 ± 4.02	7.47 ± 3.24	0.200	6.10 ± 4.30	4.59 ± 3.64	0.278
Smoking	9.25 ± 6.70	7.62 ± 3.16	0.890	6.75 ± 7.13	4.68 ± 3.51	0.715
Known lung disease	14.00 ± 4.58	7.44 ± 3.08	0.016	11.33 ± 5.50	4.51 ± 3.43	0.023
Coronary artery disease	11.33 ± 6.65	7.55 ± 3.17	0.210	8.66 ± 7.37	4.63 ± 3.51	0.226
Fever	8.00 ± 3.24	7.60 ± 3.47	0.503	5.15 ± 3.81	4.66 ± 3.75	0.633
Cough	8.52 ± 3.91	7.28 ± 2.97	0.278	5.68 ± 4.49	4.32 ± 3.23	0.343
Respiratory distress	7.30 ± 3.09	7.81 ± 3.47	0.686	4.76 ± 3.53	4.81 ± 3.82	0.958
Phlegm	8.25 ± 4.03	7.68 ± 3.38	0.782	5.50 ± 4.79	4.76 ± 3.72	0.821
Headache	7.25 ± 2.37	7.77 ± 3.50	0.956	4.25 ± 2.60	4.87 ± 3.88	0.963
Nasal discharge	6.25 ± 1.48	7.90 ± 3.52	0.284	2.87 ± 1.95	5.04 ± 3.86	0.160
Joint pain	8.00 ± 2.00	7.70 ± 3.44	0.594	5.66 ± 2.51	4.76 ± 3.80	0.428
Muscle pain	7.28 ± 2.56	7.76 ± 3.48	0.907	3.71 ± 2.43	4.92 ± 3.86	0.620
Loss of taste–smell	6.54 ± 1.86	7.93 ± 3.56	0.366	3.36 ± 1.85	5.06 ± 3.95	0.348
Nausea-vomiting	11.00 ± 8.48	7.62 ± 3.23	0.575	8.50 ± 6.50	4.69 ± 3.58	0.505
Intensive care unit transfer	11.28 ± 4.68	7.32 ± 3.01	0.031	8.71 ± 4.92	4.37 ± 3.38	0.025
Survival	7.47 ± 3.23	13.33 ± 1.52	0.010	4.54 ± 3.61	10.66 ± 0.57	0.011

When the structured total CT score values and the total CT score values were compared according to the oxygen saturation and laboratory values, they were observed to be correlated based on the r and p values. The detailed results are given in Table 7.

**Table 7:** Relationship of the structured total CT score values and the total CT score values with laboratory parameters (correlation table)

	r; p	
	Structured total CT score values	Total CT score values
Oxygen saturation	− 0.364; 0.002	− 0.398; < 0.001
Hemoglobin	− 0.289; 0.015	− 0.240; 0.043
White blood cell	− 0.398; < 0.001	− 0.390; < 0.001
Absolute neutrophil count	− 0.044; 0.718	− 0.028; 0.820
Absolute lymphocyte count	− 0.371; 0.001	− 0.355; 0.002
Creatinine	0.294; 0.013	0.339; 0.004
Lactate dehydrogenase	0.515; < 0.001	0.541; < 0.001
D-dimer	0.529; < 0.001	0.515; < 0.001
High-sensivity cardiac troponin	0.375; 0.006	0.373; 0.006

## Discussion

CO-RADS is an alternative COVID-19 pneumonia classification system to the Radiology Society of North America (RNSA) classification [5, 12]. In one study, CO-RADS was even reported to have better diagnostic performance than the system recommended by RSNA [12].

In our study, a significant difference was found between the CO-RADS groups in terms of age. This significance resulted from the difference between the CO-RADS groups 1 and 5 and groups 4 and 5. Patients in CO-RADS 5 represented the oldest group. We can state that age is an important factor in the lung involvement of the disease. In a meta-analysis study, it was reported that clinical and laboratory findings showed the course of severe disease, especially in elderly patients [13]. In another study, it was determined that the disease progressed differently in different age groups [14]. This was mostly attributed to the weakness of immunity [15].

In the current study, the significant difference between the CO-RADS groups in terms of ICU transfer resulted from the comparison of the groups 1 and 3. Twenty percent of the patients in the CO-RADS group 3 were transferred to ICU. From this, we can conclude that despite their low risk of COVID-19, it is important to follow up patients in the CO-RADS group. This group should perhaps be regarded as the unstable group. Currently, there are not sufficient data on this subject in the literature, but more studies will likely be carried out in the future.

When we examined the relationship between CO-RADS and oxygen saturation, we determined that saturation was lower in cases with more lung involvement, as expected. There are new treatment options available in the literature in patients with hypoxia, and one of them is tocilizumab [16]. Although tocilizumab is an effective treatment for hypoxia, it is a risky agent in terms of its side-effect profile. In particular, the risk of hepatotoxicity has been previously reported [17, 18]. Therefore, the CO-RADS classification can be used as a parameter in determining treatment indication together with saturation values. The clinician may consider the CO-RADS classification in making a treatment decision.

Considering the relationship between CO-RADS and creatinine, significant differences were observed between the CO-RADS groups 1 and 5 and groups 4 and 5. Creatinine may be an important marker in lung involvement, which is known to be associated with kidney functions. In one study, creatinine values were found to be significantly higher in critically ill patients who were hospitalized [19], which is consistent with our study.

In relation to LDH, we observed significant differences between the CO-RADS group 3 and 5, groups 4 and 5, and groups 1 and 5. The highest LDH value was in the CO-RADS 5 category, and therefore LDH can distinguish this group from all the other categories. LDH can be considered as an important marker in parenchymal involvement, especially in the presence of bilateral and diffuse involvement. In particular, LDH values ​​above 200 can support CO-RADS 5. Since LDH is a parameter associated with tissue damage, it can be stated that tissue damage is more intense in cases classified as CO-RADS 5. We consider that LDH can be used to predict the long-term outcomes of patients in this group.

When the relationship between CO-RADS and D-dimer was analyzed, there were significant differences between the CO-RADS groups 3 and 5 and groups 1 and 5. The D-dimer level was the highest in the CO-RADS group 5. From this, we can conclude that one of the causes of diffuse lung involvement is the tendency to clot. In a meta-analysis study, lymphopenia, thrombocytopenia and high CRP, LDH and D-dimer values ​​were found to be associated with advanced disease [20]. However, to our knowledge, there is no other that has compared the laboratory tests of patients according to their CO-RADS groups. In this respect, our study makes a contribution to the literature.

The hs-cTnT level was the highest in the CO-RADS group 5 compared to the remaining groups. Although our study did not evaluate the myocardial involvement of COVID-19, this result suggests that patients in the CO-RADS 5 group are at higher risk of cardiac involvement.

The highest CRP value was also observed in the CO-RADS group 5. Previous studies showed that elevated CRP levels were generally correlated with the severity of the disease at the time of detection and lung lesions [21, 22]. Therefore, our CRP results are in agreement with the literature.

In our study, we also grouped the parenchymal involvement of the patients in terms of ground glass, mixed and consolidation patterns, since we consider that the CT pattern should have a place in the scoring system. For this purpose, we obtained the structured total CT score value in the regression analysis performed with the total CT scoring [7] and the scores obtained from the pattern groups. In our study, when the total CT score and the structured total CT score values were compared according to the CO-RADS groups 3, 4 and 5, significant differences were found between the groups 3 and 5 in terms of the structured total CT score and between the groups 3 and 5 and groups 4 and 5 in terms of the total CT score value. In a previous study, scoring was performed according to < 25% lung involvement, mild; 25–50% involvement, moderate; and > 50% involvement, advanced [23]. In our study, scoring was undertaken separately for each lung lobe, and therefore we consider this to be an innovative feature of our study since we obtained a structured total CT lung score by taking into account the pattern groups.

In another study using the scoring system utilized in our study, each lobe was evaluated separately, and then the total score of the whole lung was found to be positively correlated with RT-PCR positivity and male gender [24]. In our study, no significant difference was found in terms of gender.

When the relationship of creatinine with the pattern groups was examined, creatinine was found to significantly differ between the normal lung parenchyma (pattern 0) and the ground glass pattern (pattern 1) groups. In addition to showing the extent of the disease, creatinine is also an effective parameter in showing the intensity of lung involvement. When the pattern groups were compared according to the total CT score values, no significant difference was observed. In a similar study conducted with 165 patients to evaluate lung involvement pattern groups, the CT patterns were divided into groups 0–4, representing normal findings, bronchopneumonia, organizing pneumonia, progressive organizing pneumonia, and diffuse alveolar damage [25]. In our study, the pattern groups ranged from 0 to 3, and the CT pattern was categorized according to the density of the lesions in the lung. We consider that our own classification is more applicable and practical in daily clinical practice.

In a previous study, mortality was found to be significantly higher in patients with a higher total CT score value at the time of initial diagnosis [26]. In another study, the total CT score value was found to be associated with requirement of hospitalization, requirement of intensive care unit, and one-month mortality [27]. In our study, when the structured total CT score and the total CT score values were compared in terms of ICU transfer, survival, oxygen saturation and laboratory values, they were observed to be correlated with each other. The comparison of many parameters revealed that the structured total CT scoring system provided similar results to the total CT scoring system. Structured scoring may even be regarded a more accurate scoring system because it also includes the lung involvement pattern.

One of the limitations of our study is that we only included RT-PCR-positive patients. The alternative diagnosis group followed up in the CO-RADS category 2 was excluded. In addition, since we did not encounter pattern 3 in any of our patients, we did not include this pattern in our evaluations.

## Conclusions

In conclusion, creatinine can be considered as an important marker for the CO-RADS and pattern classifications in lung involvement. LDH can be regarded an important marker of parenchymal involvement, especially in the presence of bilateral and diffuse involvement. The presented structured total CT scoring system is a new system that can be used as an alternative to total CT scoring.

## Data Availability

All data generated or analysed during this study are included in this published article.
